# Insights Into Dynamics of Inhibitor and Ubiquitin-Like Protein Binding in SARS-CoV-2 Papain-Like Protease

**DOI:** 10.3389/fmolb.2020.00174

**Published:** 2020-08-04

**Authors:** Yuliana K. Bosken, Timothy Cholko, Yuan-Chao Lou, Kuen-Phon Wu, Chia-en A. Chang

**Affiliations:** ^1^Department of Chemistry, University of California, Riverside, Riverside, CA, United States; ^2^Biomedical Translation Research Center, Academia Sinica, Taipei, Taiwan; ^3^Institute of Biological Chemistry, Academia Sinica, Taipei, Taiwan

**Keywords:** structure-based drug design, computational biology and chemistry, non-covalent molecular recognition, conformational change, protein entropy, biophysics, protein-protein interaction, protein inhibition

## Abstract

Covid-19 is caused by a novel form of coronavirus for which there are currently no vaccines or anti-viral drugs. This virus, termed SARS-CoV-2 (CoV2), contains Papain-like protease (PLpro) involved in viral replication and immune response evasion. Drugs targeting this protease therefore have great potential for inhibiting the virus, and have proven successful in older coronaviruses. Here, we introduce two effective inhibitors of SARS-CoV-1 (CoV1) and MERS-CoV to assess their potential for inhibiting CoV2 PLpro. We ran 1 μs molecular dynamics (MD) simulations of CoV2, CoV1, and MERS-CoV ligand-free PLpro to characterize the dynamics of CoV2 PLpro, and made comparisons between the three to elucidate important similarities and differences relevant to drug design and ubiquitin-like protein binding for deubiquitinating and deISGylating activity of CoV2. Next, we simulated the inhibitors bound to CoV1 and CoV2 PLpro in various poses and at different known binding sites to analyze their binding modes. We found that the naphthalene-based ligand shows strong potential as an inhibitor of CoV2 PLpro by binding at the putative naphthalene inhibitor binding site in both computational predictions and experimental assays. Our modeling work suggested strategies to improve naphthalene-based compounds, and our results from molecular docking showed that the newly designed compounds exhibited improved binding affinity. The other ligand, chemotherapy drug 6-mercaptopurine (6MP), showed little to no stable intermolecular interaction with PLpro and quickly dissociated or remained highly mobile. We demonstrate multiple ways to improve the binding affinity of the naphthalene-based inhibitor scaffold by engaging new residues in the unused space of the binding site. Analysis of CoV2 PLpro also brings insights into recognition of ubiquitin-like proteins that may alter innate immune response.

## Introduction

Covid-19, caused by a novel form of coronavirus, has created a global health crisis due to the lack of vaccines and anti-viral drugs. Over the past two decades, coronaviruses such as the Severe Acute Respiratory Syndrome coronavirus (SARS-CoV-1 or CoV1) and Middle East Respiratory Syndrome coronavirus (MERS-CoV) have caused mass human fatality. In late 2019, the novel form of coronavirus, known as SARS-CoV-2 (CoV2), spread rapidly from Wuhan, China to all continents of the world within months, causing widespread mortality and worldwide panic (Centers for Disease Control and Prevention, 2020). The only way to curtail the spread of the virus thus far has been through strict, indefinite quarantine of millions of people. Clearly, development of anti-viral drugs capable of inhibiting CoV2 is of paramount importance.

**FIGURE 1:**
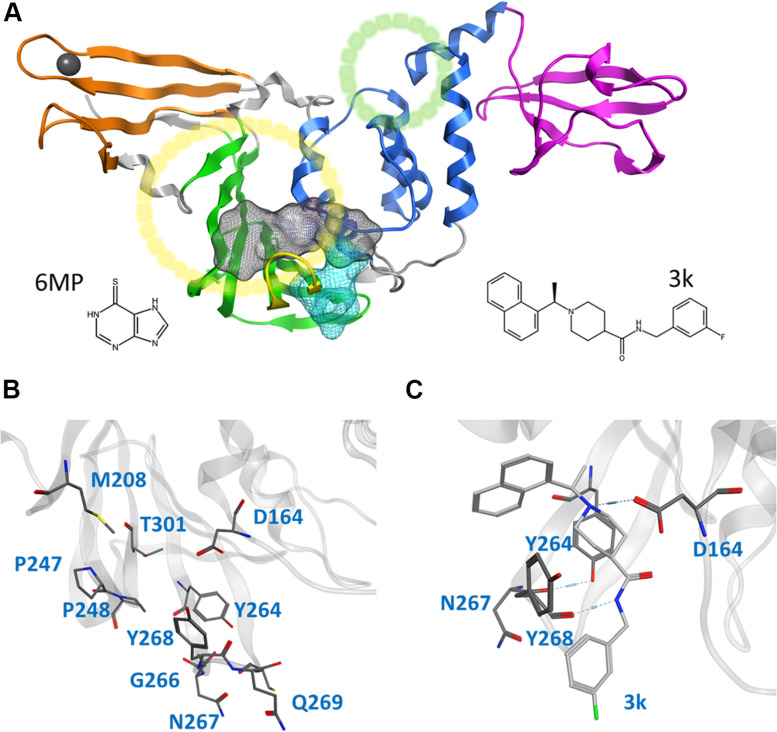
Cartoon representation of the entire CoV2 PLpro structure and close-ups of regions important to ligand binding. **(A)** PLpro with the four domains and other major regions indicated as follows: fingers, orange; palm, green (BL2 loop, yellow); thumb, blue; UBL, magenta; SUb1 and SUb2, yellow and green circles, respectively. The putative 3k binding site is shown as a gray surface and the active site as a teal surface. 6MP was docked to the putative 3k site and active site. **(B)** Important binding site residues. **(C)** 3k (light gray) engaging in hydrogen bonds with D164 and Y268, and the important BL2 loop-stabilizing hydrogen bond between Y264 and N267.

CoV2 contains a Papain-like protease (PLpro) that is vital for viral replication (Harcourt et al., 2004). PLpro is responsible for the proteolytic processing of the product of open reading frame 1a (ORF1a) in the replicase gene of CoV2, a large viral polyprotein containing non-structural proteins which form the replicase complex (Wertz and Murray, 2019). PLpro exists as a monomer in biological settings and has the USP fold, typical for the ubiquitin-specific proteases (USP) family in humans, which is topologically organized into four domains – UBL, thumb, palm, and fingers (Ye et al., 2009; Figure 1A). The peptide bond cleavage in the active site is catalyzed by a conserved catalytic triad comprised of residues Cys111, His272, and Asp286 (Baez-Santos et al., 2015). In addition, PLpro possesses deubiquitinating and deISGylating capabilities (Sulea et al., 2005) which interfere with critical signaling pathways leading to the expression of type I interferons, resulting in antagonistic effect on host innate immune response (Devaraj et al., 2007; Bekes et al., 2016). Therefore, inhibition of PLpro activity can halt viral replication and disrupt its role in host immune response evasion, making it an excellent anti-viral drug target.

CoV2 PLpro exhibits a high sequence similarity to CoV1 PLpro (Supplementary Figure S1); in particular, the binding site and active site residues are nearly identical. We have introduced a leading naphthalene-based inhibitor, 3k, and chemotherapy agent 6-mercaptopurine (6MP) (Figure 1A), which successfully inhibited CoV1 PLpro and MERS-CoV PLpro, respectively (Chou et al., 2008; Baez-Santos et al., 2014; Cheng et al., 2015), to assess their binding affinity for CoV2 PLpro. The 3k binding site is adjacent to the catalytic triad and sterically inhibits the binding of ubiquitin (Ub) and Interferon-stimulated gene 15 (ISG15) by occupying the space normally reserved for their C-terminal (LXGG cleavage site) at ubiquitin binding subsite 1 (SUb1) (Figure 1A). Compounds capable of binding to this site therefore exhibit high inhibitory capabilities.

In this work, we carried out several molecular dynamics (MD) simulations of ligand-free and ligand-bound CoV2, CoV1, and MERS-CoV PLpro (Table 1). Based on detailed examination of the CoV2 3k binding site, we provide guidance and suggestions for optimization of compounds targeting this site. Moreover, by simulating 3k bound to CoV2 and CoV1 PLpro, we show that it exhibits a highly similar binding mode in both proteins, suggesting that 3k and similar compounds should have an inhibitory effect on CoV2 PLpro. After analysing the binding mode and binding site, we constructed and docked new ligands based on the 3k scaffold which showed improved binding affinity over the current molecule. Additionally, we carried out experimental assays to validate 3k binding to CoV2 PLpro and inhibit enzymatic function. We show that the overall dynamics of ligand-free PLpro in all analyzed systems is highly similar, with comparable flexibility in BL2 loop, zinc-binding region and UBL domain. Our detailed description of 3k binding in the protein provides insight into the essential interactions necessary for successful fragment-based drug design. Additionally, we provide well-sampled dynamics of the available CoV2 PLpro crystal structures for wider use as a guide to potential drug binding sites or in docking and drug screening studies.

**TABLE 1:** Summary of all simulations performed.

**Simulation index**	**PDB**	**Protein system**	**Length**
**Summary of simulations**			
MD1 (1, 2)	6W9C	CoV2 PLpro	1 μs, 500 ns
MD2 (1, 2)	6WRH	CoV2 PLpro	1 μs, 500 ns
MD3 (1, 2)	4OW0	CoV1 PLpro	1 μs, 500 ns
MD4 (1, 2)	4RNA	MERS-CoV PLpro	1 μs, 500 ns
MD5a (1–3)	6W9C	CoV2 PL pro complexed w/3k (pose A)	1 μs, 500 ns, 200 ns
MD5b (1–3)	6W9C	CoV2 PL pro complexed w/3k (pose B)	3 × 200 ns
MD5c (1–3)	6W9C	CoV2 PL pro complexed w/3k (pose C)	3 × 200 ns
MD5d (1–3)	6W9C	CoV2 PL pro complexed w/3k (pose D)	3 × 200 ns
MD6 (1–3)	4OW0	CoV1 PLpro complexed w/3k	1 μs, 500 ns, 200 ns
MD7a (1–3)	6W9C	CoV2 PLpro complexed w/6MP (in putative site)	3 × 200 ns
MD7b (1–3)	6W9C	CoV2 PLpro complexed w/6MP (in active site)	3 × 200 ns

**All ligand-free proteins were simulated twice under identical conditions except for the initial random number seed, first for 1 μs, followed by a 500 ns secondary run to confirm consistency in the observed dynamics. Similarly, all ligand-bound proteins were simulated three times for at least 200 ns. Where necessary, secondary and tertiary runs are referred to by a dash and number after the main designation, e.g., MD1-2 means the second run of simulation MD1. These trajectories are available on our group webpage: http://chemcha-gpu0.ucr.edu/software/ and the COVID -19 Molecular Structure and Therapeutics Hub: https://covid.molssi.org/.**

## Results and Discussion

We analyzed 1 μs trajectories of ligand-free CoV2, CoV1, and MERS-CoV PLpro to uncover the overall protein dynamics of the novel coronavirus protease and to make comparisons to older conronavirus PLpro for which inhibitors have been developed. In addition, we simulated ligand-bound trajectories of CoV1 and CoV2 PLpro to assess potential effectiveness of one naphthalene-based and one thiopurine inhibitor – 3k and 6MP, respectively – in the 2019 coronavirus. We showed that 3k formed stable interactions with CoV2 PLpro, suggesting that the compound can bind to the protein, which was verified by experimental assays. Moreover, we designed and docked new ligands based on the 3k-scaffold to CoV2 PLpro, and show the they achieve improved binding affinity. Protein flexibility, entropy, and conformational changes were analyzed in the ligand-free protein simulations to characterize the overall protein dynamics and to assess similarities and differences relevant to inhibitor or Ub binding in CoV2 PLpro. The ligand-bound MD simulations were analyzed for a detailed characterization of ligand binding modes by analysing residue-wise interactions, binding energy, and ligand-induced conformational changes.

### Structure and Dynamics of Ligand-Free CoV2 PLpro and Implications for Drug Discovery

Dynamic regions of potential importance to small molecule drug or Ub binding in CoV2 PLpro include portions of the thumb domain (containing SUb2), the fingers region (adjacent to SUb1) and the BL2 loop (directly adjacent to the 3k binding site). Principal component analysis (PCA) shows that the dominant overall motion of CoV2 PLpro occurs due to high flexibility of the fingers domain – especially the zinc-binding region, the BL2 loop, and the UBL domain (Supplementary Figure S2). The fingers domain is the most mobile region of PLpro, and has been shown to crystallize in different conformations (Baez-Santos et al., 2015). Because this region is highly flexible and challenging for a small molecular inhibitor to bind tightly, it is not considered as an ideal druggable site.

This study focuses on the binding site of naphthalene inhibitors (Figure 1A), a druggable site reported in previous studies (Baez-Santos et al., 2014) that is directly adjacent to the PLpro active site to prevent off-target binding to the highly similar active site of human proteins (Kemp, 2016). Flexibility of the BL2 loop, which can result in an open or closed conformation, indicates potential of this binding site to accommodate compounds with new scaffolds or different derivatives of 3k, which may include larger substitutions to strengthen binding with underutilized regions. One such region is the hydrophobic portion lined by residues Met208, Pro247 and Pro248 (Figure 1B). Closer to the BL2 loop, Gly266 may be able to provide inhibitor binding specificity through hydrogen bond formation. The portion of the binding site extending just past the BL2 loop in the direction of the UBL domain presents substantial space to engage PLpro residues with larger ligands (Figure 1B).

**FIGURE 2:**
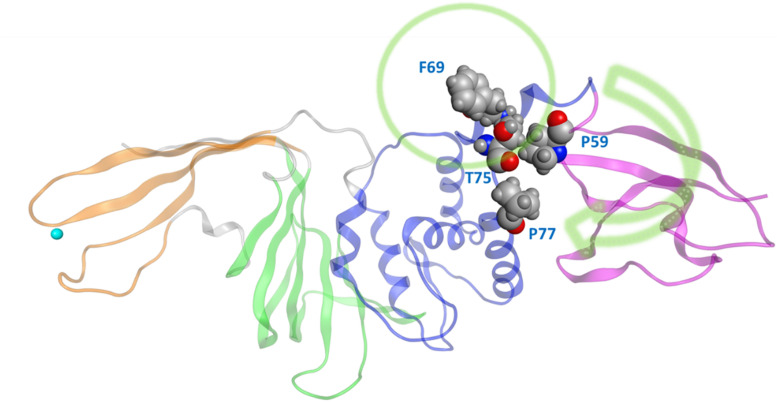
The movement of UBL in CoV2 allows for interactions between UBL residue Pro59 and the thumb domain residues Thr75 and Pro77. These interactions subsequently result in different rotameric states for the nearby key Ub-interacting residue Phe69/Phe70 in CoV2/CoV1. Green arrow indicates the major motion of UBL; green circle indicates SUb2.

One very prominent motion of CoV2 PLpro is partial rotation of the UBL domain and its relative position to “ridge” helix (Asp62 – His73) in the SUb2 region (Figure 2). The function of the UBL domain is unknown, and although some studies suggest that it has no effect on function of PLpro (Clasman et al., 2017), we observed one noteworthy interaction involving this domain. Transposition of UBL toward the thumb domain results in hydrophobic interactions between Pro59 of the UBL domain and Pro77 and Thr75; Thr75 then interacts with Phe69 of the “ridge” helix and can alter the latter residues conformation. Mutating this Phe was shown to affect the binding affinity of ISG15 and K48 linked diUb in CoV1 PLpro (Ratia et al., 2014), so the conformational dynamics of this residue may also be important in CoV2. Since CoV1 exhibits this same interaction between UBL residues and this Phe residue, we compared the conformation populations for Phe69/70 (residue numbering differs by 1 between Cov2/CoV1) between the two PLpros. Notably, Cov1 contains Leu at position 75 (rather than Thr75 as in Cov2) and its concerted motion with Phe70 yields four different conformations. The Phe69-Thr75 interaction in CoV2 affects Phe69 to a lesser extent, resulting in just two distinct conformations of the same sidechain (Supplementary Figure S3). In contrast to the dynamic SUb2 region, SUb1, the binding site for distal Ub, does not show any significant structural fluctuation. Ub-interacting hydrophobic residues Met208 and Pro247 are exposed to the solvent to potentially engage in ligand interactions (Supplementary Figure S4), which may be an alternative method to disrupt Ub binding at SUb1 in aside from blocking its C-terminal from the LXGG cleavage site.

**FIGURE 3:**
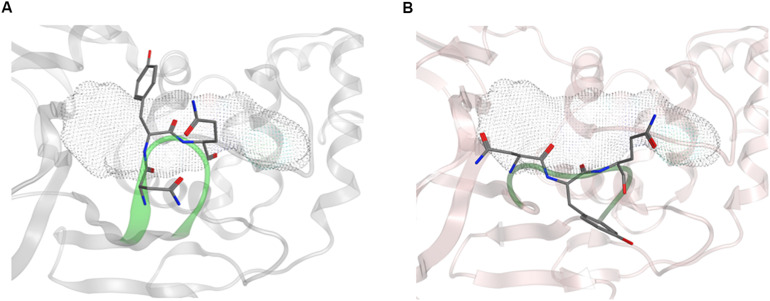
Conformation of Asn267 and Tyr268, 3k binding site is indicated with dotted surface. **(A)** Common conformation of these residues observed in CoV2 (6WRH) and CoV1 simulations. **(B)** Unique conformation observed only in CoV2 (6W9C) simulation.

**FIGURE 4:**
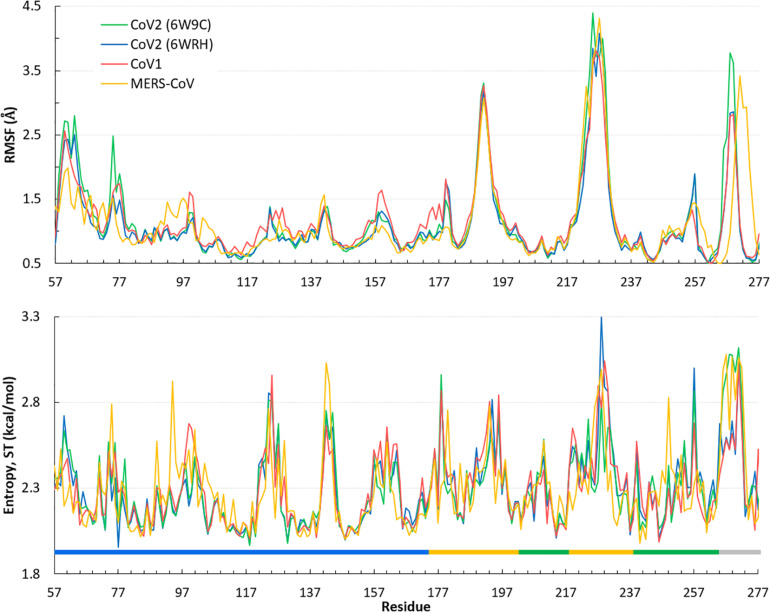
Quantifying the overall dynamics and conformational flexibility of PLpro. Top: RMSF of alpha C atoms over the 1 μs trajectory of all four ligand-free PLpros. The spike at the BL2 loop (∼ residues 265–275) is larger for CoV2 (6W9C) and MERS-CoV because of their more open conformations during all or part of the simulations. Residues 1–56 (UBL domain) and 300–311 (C-terminal) have been omitted for clarity. Bottom: Dihedral entropy of the psi angle for CoV2, CoV1, and MERS-CoV systems. PLpro regions indicated by color bar: thumb, blue; fingers, yellow; palm, green; BL2 loop, gray. Entropy calculated at 298 K.

Overall, the dynamics of the CoV2, CoV1, and MERS-CoV ligand-free PLpro is quite similar. Supplementary Figure S2 shows the first principal component of overall motion for all three systems, which reveals similarly high mobility in the zinc-binding domain, BL2 loop and UBL domain. CoV2 simulations MD2 and MD3 showed similar flexibility to CoV1 in most of the highly flexible regions during the entire course of the 1 μs MD simulations. Notably, in MD1, the initial crystal structure conformation shows a unique conformation of Asn267 and Tyr268 (Figure 3), resulting in larger root-mean-square fluctuation (RMSF) and dihedral entropy values than those computed for the other ligand-free PLpros (Figure 4), as well as additional rotameric states (Supplementary Figure S5). Around 420 ns into MD1-1 and just 20 ns into MD1-2 the residues change conformation to ones highly similar to those in MD2 (CoV1) and MD3 (CoV2), at which point the RMSFs become nearly identical (Supplementary Figure S6). This unique conformation of key ligand-binding residue Tyr268 (Chaudhuri et al., 2011) is not preorganized for protein-ligand complex formation, thus it may incur a cost in conformational energy or entropy which can affect inhibitor binding. Supplementary Figure S7 compares backbone dihedral angle populations of several binding pocket residues between CoV1 and CoV2 PLpro over the simulation time. In terms of dihedral entropy as well, CoV1 and CoV2 are quite similar. The entropy calculations for the backbone torsion show only a few regions with higher conformational sampling in CoV2, mainly in the zinc binding region of the fingers domain and BL2 loop (Figure 4). In MERS-CoV, the amino acid composition of the BL2 loop is entirely different from CoV2 with the exception of two flanking Gly residues (Lee et al., 2015). Although the entropy and RMSF show similar flexibility of the loop, its overall conformation relative to the palm domain is more open than in CoV1 and CoV2. BL2 remains in this open conformation, which appears to be stabilized by hydrophobic interactions of Gln270, Glu273, Thr274, and His278 sidechains, for the entirety of MD4 (Supplementary Figure S8).

### Comparison of Ligand-Free and Ligand-Bound Structures CoV1 and CoV2 PLpro

Revealing detailed protein conformational changes after ligand binding provides insight to the key binding interactions relevant to drug development. We compared ligand-free and ligand-bound systems to identify how binding shifts the populated conformations of surrounding residues.

**FIGURE 5:**
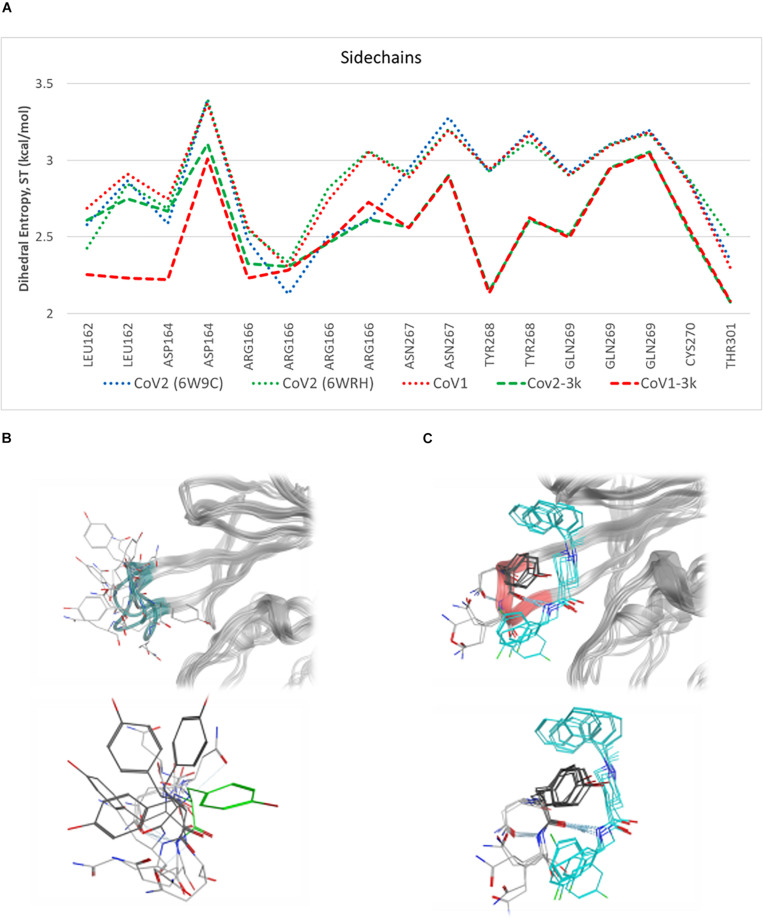
Plot of entropy for 3k binding site residues and pictures of their conformations. **(A)** Sidechain dihedral angle entropy for 3k binding site residues in ligand-free and 3k-bound CoV1 and CoV2 PLpro shows the stabilization of these residues after ligand binding. **(B)** An overlay of several MD frames shows the range of conformations adopted by BL2 loop (dark green) residues in the ligand-free state. Tyr268 in the ligand-bound conformation is shown in light green. **(C)** The conformational sampling of these residues is dramatically reduced upon binding of 3k (teal). Entropy calculated at 298.

**FIGURE 6:**
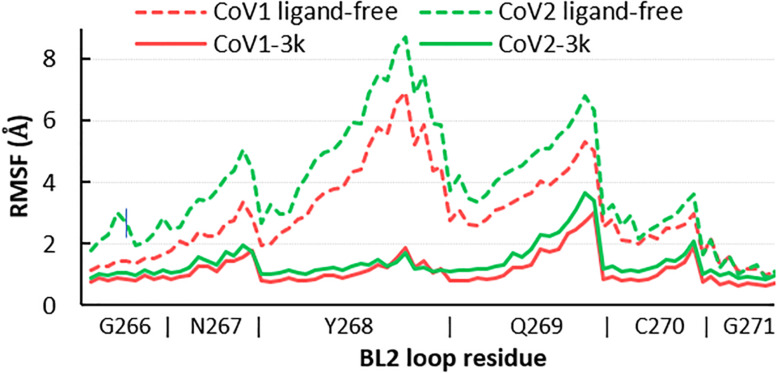
RMSF of all atoms in the BL2 loop in ligand-free and 3k-bound CoV1 and CoV2 PLpro. In both systems, ligand binding induces a closed, ordered BL2 loop conformation resulting in dramatically reduced mobility of this region. The x-axis indicates the range of atoms in each BL2 loop residue.

Simulations show the CoV2 PLpro BL2 loop having significant flexibility in ligand-free proteins. Residues Asn267, Gln269, and most importantly Tyr268, account for most of this motion, which resembles opening and closing of the loop (Figures 5A,B). MD5a-d all show that the BL2 loop in CoV2 PLpro is highly stabilized by ligand binding, as most residues interacting with the ligand are confined to a single conformation (Figure 5C). Most notably, the sidechain and backbone rotation of key residue Tyr268 is minimized through a hydrogen bond and strong vdW interactions with the ligand, as detailed in next subsection. The very same ligand-induced stabilization of the BL2 loop is seen for CoV1 PLpro (Figure 6). The central portion of this binding pocket, which houses the piperidine, carboxyl and amide moieties of 3k, is narrower and may already be maximized in terms of inhibitor binding potential. Two key hydrogen bonds form here (Figure 1C), and it has been shown that substitutions larger than a methyl group or hydrogen at the benzylic-naphthyl or benzyl position, respectively, on naphthalene inhibitors lowered their effectiveness (Baez-Santos et al., 2014).

Residues involved in consistent interactions with the ligand show a significant difference in dihedral entropy. Hydrogen bonds formed with 3k substantially restrict conformational exchange for the associated residues. Asp164 and Tyr268 appear to be a key aspect in the 3k-CoV2 PLpro interactions, which is reflected by the decreased dihedral entropy (Supplementary Figure S9).

Comparison of MD5a-d to MD6 (Table 1) reveals that 3k binds very similarly in CoV2 and CoV1 PLpro, inducing a closed, ordered conformation of the BL2 loop around the ligand. Moreover, the RMSD values of 3k over 200 ns in MD5a and MD6 of 1.06 and 0.95 Å, respectively, reveal similar stability in the CoV2 and CoV1 putative binding sites. The naphthalene moiety occupies the hydrophobic cleft of the pocket and the fluorophenyl ring protrudes from the opposite end of the pocket while retaining a high degree of mobility relative to the rest of the compound. The high similarity of these binding modes indicates strong potential of naphthalene inhibitors to have an inhibitory effect on CoV2 PLpro through a similar mechanism as in CoV1 PLpro.

### Ligands Binding Modes in CoV2 PLpro and Strategies for Drug Design

**FIGURE 7:**
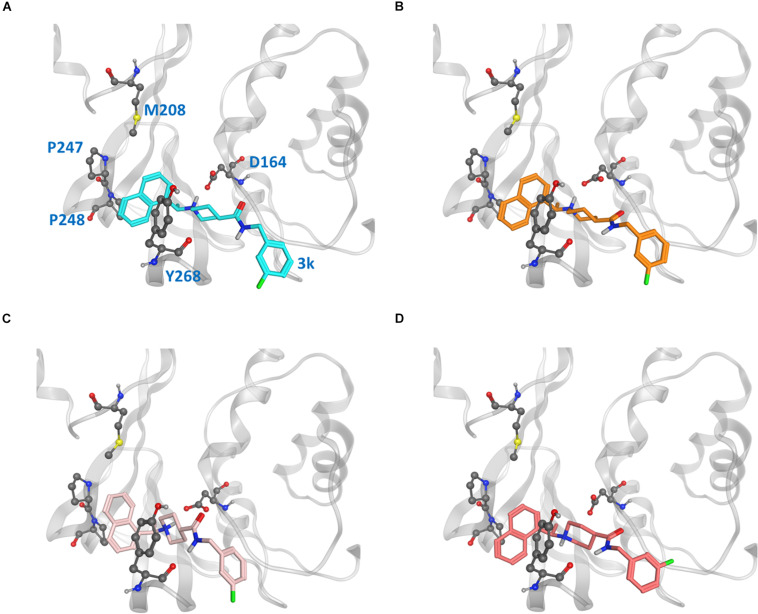
Ligand poses **(A–D)** from which the CoV2 PLpro-3k complex simulations began. Key binding site residues are shown in gray to show the difference in relative orientation of the ligand in each pose.

Because the putative naphthalene inhibitor binding site of CoV2 PLpro is comprised by the same residues as in CoV1 PLpro, we examined one of the most effective second generation naphthalene inhibitors of CoV1 PLpro (Baez-Santos et al., 2014), 3k, to reveal structural information regarding binding to CoV2 PLpro for future structure-based drug development. After analysing free and ligand-bound CoV1 PLpro simulations, we docked 3k to one CoV2 PLpro conformation to obtain four different binding poses (Figure 7) and ran three simulations for each pose (MD5a-d). Poses A and B were nearly the same, except B was docked with unconstrained side chain rotations allowed, so 3k starts slightly rotated with respect to A. MD5c and MD5d start with a 180° rotation of the naphthalene or piperidine moiety, respectively, compared to MD5a. Ultimately, MD5a, b and d all establish the same major interactions with PLpro. The majority of our discussion focuses on MD5a, where the initial conformation (Figure 7A) is the most similar to the CoV1 PLpro-3k crystal structure. Our results indicated that 3k binds strongly and suggest that the ligand can inhibit the enzymatic function of CoV2 PLpro. Experimental results have confirmed 3k binding by showing the NMR spectrum for ligand-free and -bound CoV2 PLpro (Supplementary Figure S10).

**FIGURE 8:**
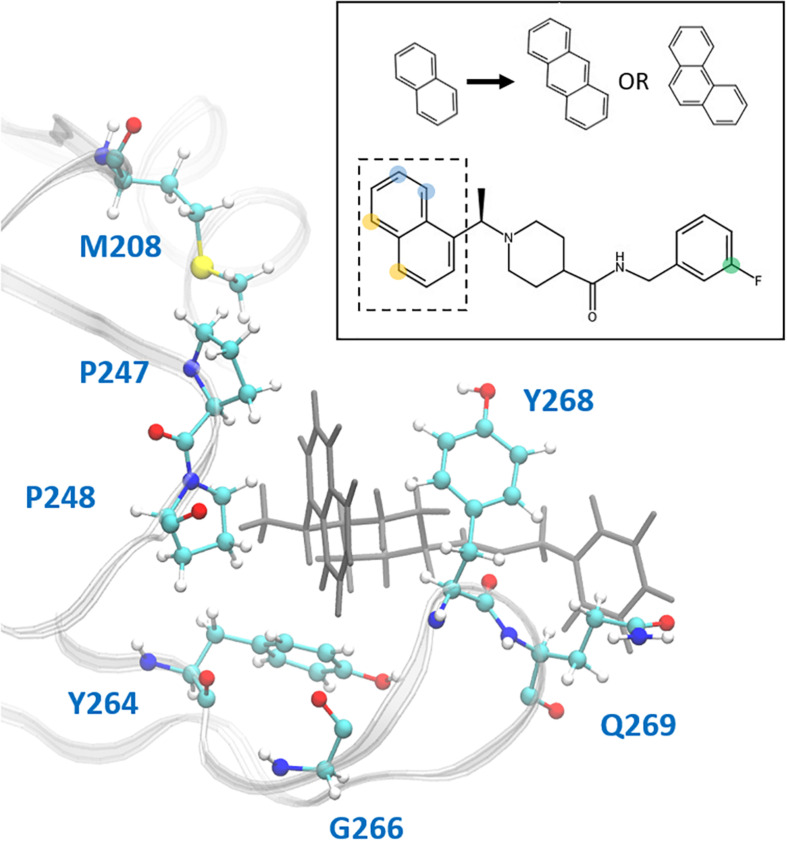
Ligand 3k (gray) in the CoV2 PLpro binding site. Residues with which new interactions are achievable or current ones can be strengthened are labeled and shown in ball-and-stick representation. Top-right: 2D molecular structure of ligand 3k indicating proposed substitution positions for increased binding affinity. Substitutions at the yellow positions may be capable of additional hydrophobic contacts with Pro247, Pro248, or Met208. Substitutions at blue positions may be capable of additional hydrophobic contacts with Pro248 or Tyr264, or hydrogen bonds with the backbone carboxyl of Gly266. Finally, substitutions at the green position in combination with an extended benzene linkage may be capable of increased attractive interactions with Gln269 or other nearby residues. The naphthalene moiety is indicated by the dashed box, with the proposed anthracene or phenanthrene substitutions indicated above.

In the hydrophobic portion of the binding site, the naphthalene moiety sits stably between residues Pro247 and Pro248 to one side and Tyr268 on the other (Figure 8) However, additional space exists in this pocket to engage more residues. Specifically, it may be possible to increase the hydrophobic interactions here with a methyl (or larger) substitution on the naphthalene to further engage in vdW interactions with Pro248 or Tyr264 (Figure 8, blue dots). Pro248 and Met208 can also be further engaged in hydrophobic interactions with substitutions at the appropriate positions on naphthalene (Figure 8, yellow dots), or potentially even a substitution of the entire naphthalene moiety for a larger aromatic structure such as anthracene or phenanthrene (Figure 8). MD5c (Figure 7C), in which the naphthalene in the initial 3k conformation is flipped 180° relative to MD5a, provides support for this idea, as the flipped moiety is seen making closer contact with residues Met208 and Pro248, resulting in greater attraction to these residues (Supplementary Figure S11) and slightly lower overall binding energy (Table 2) than in MD5a. Lastly, a hydrogen bond donor or acceptor substitution at the correct naphthalene position (Figure 8, blue dots) may be able to engage with the Gly266 backbone.

**TABLE 2:** MM/PBSA energy breakdowns for the binding energy from simulations of the four different starting poses (A–D) of CoV2 PLpro-3k and CoV1 PLpro-3k.

	**CoV1-3k**	**CoV2-3k A**	**CoV2-3k B**	**CoV2-3k C**	**CoV2-3k D**
**MM/PBSA binding energies**					
ΔE_*elec*__+__*PB*_	11.9	11.8	11.6	11.0	14.7
ΔE_*vdW*__+__*np*_	–28.9	–26.2	–28.1	–28.7	–27.0
ΔE_*MM/PBSA*_	−17.0 ± 3.9	−14.5 ± 4.3	−16.5 ± 4.5	−17.7 ± 3.6	−12.3 ± 5.4

**ΔE_*elec*__+__*PB*_ is the electrostatic plus polar solvation energy contributions, and ΔE_*vdW*__+__*np*_ is the van der Waals plus non-polar solvation energy contribution. ΔE_*MM/PBSA*_ is the binding energy predicted by MM/PBSA. Energies are in kcal/mol; values are ± SD.**

3k engages in two strong hydrogen bonds with the protein: one to Asp164 and the other to the backbone of Tyr268. Notably, even in MD5d, which began with the piperidine nitrogen and its hydrogen pointed in the opposite direction of Asp164, the entire moiety rotates after 25–140 ns (varying between the three runs) to establish the hydrogen bond with this residue. Previous studies found that bulky ligand substitutions that occupy this portion of the pocket decreased inhibition^1^. A possible explanation is that the specific ligand orientation needed to maintain both of these strong hydrogen bonds was not attainable due to the additional bulk. Moreover, we observed a consistent intra-protein hydrogen bond between Tyr264 and Asn267 in ligand-bound CoV2 that could be disrupted by larger ligand substitutions here, which may destabilize the closed BL2 loop. Indeed, analysis of this interaction shows very high correlation between formation of the hydrogen bond and a closed loop conformation (Supplementary Figure S12). A small hydrophobic pocket formed by Tyr264, Tyr273, and Thr301 accommodates the methyl group at the benzylic-naphthyl position.

The fluorophenyl ring of 3k appears to interact mostly with the hydrocarbon portion of Gln269, but also engages in vdW interactions with Tyr268 (Figure 8). However, because of the openness of PLpro in this region, the position of the ring fluctuates widely, and it rotates freely with the fluorine observed at several positions consistent with 360-degree rotation. Increasing ligand engagement with PLpro residues is achievable in this region, although previous attempts at doing so in CoV1 PLpro had mixed results. Substitutions on the benzyl ring in first generation naphthalene inhibitors found that anything bulkier than methyl at the ortho position decreased inhibition (Baez-Santos et al., 2014), however, the linkage between the amide and benzene ring was one carbon shorter than in the second generation, possibly causing the added bulk to disrupt one of the two important hydrogen bonds with Tyr268 or Asp164. With a longer linkage to the benzene in second-generation naphthalene inhibitors, various benzene substitutions were tested, but showed no clear trend in effectiveness. Ultimately, the fluorine substitution at the meta position, as seen in 3k, showed the best result. Extending the linkage between the amide and benzene ring by one additional carbon was found to weaken inhibition, providing evidence that benzene ring primarily contributes to binding through vdW interactions with Tyr268 and Gln269, and so needs to be close to those residues. This is consistent with our observations and residue-wise force calculations as well (Supplementary Figure S11).

One method to increase binding affinity in this region may be through increasing the hydrophobic surface area of the benzyl end of the ligand. This can be achieved either through substitution of methyl or larger hydrocarbon groups onto the benzene ring, or by replacing the benzene with a bulkier group, such as naphthalene. Although, as previously stated, it has been shown that both increasing ligand bulkiness near the benzene end and extending the linkage to benzene can sometimes lead to decreased inhibition, changing these two factors simultaneously has not been tested. A longer linkage may accommodate increased bulk, while the added hydrophobic mass can still reach residues Tyr268 and Gln269 for attractive interactions. Moreover, since no clear trend in ligand effectiveness from substitutions on the benzyl ring has been found, we suggest exploration of the available space in this portion of the binding site.

**FIGURE 9:**
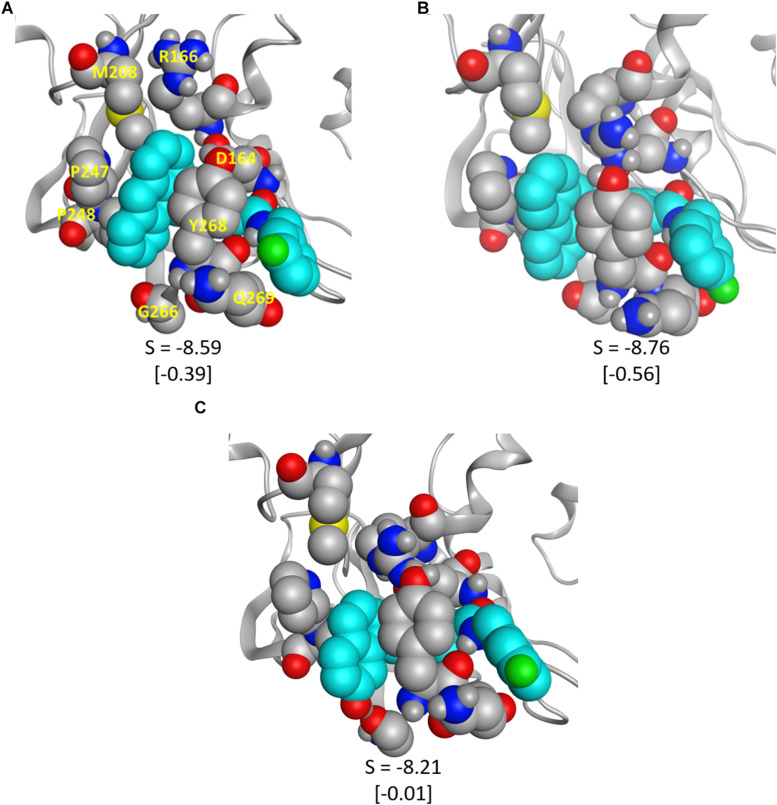
The modified ligands (teal) docked to CoV2 PLpro. **(A)** 3k with anthracene substituted for naphthalene, **(B)** 3k with phenanthrene substituted for naphthalene, and **(C)** 3k with a hydroxyl substituted on the napthyl moiety; the hydrogen bond with Gly266 is clearly visible. S is the docking score given by *MOE*, and the difference between the pictured docked conformation and the best 3k score is show in brackets.

To validate some of our proposed modifications to the current naphthalene-based scaffold, we docked these modified ligands to the same CoV2 PLpro conformation used to dock 3k. First, to investigate the potential for making additional hydrophobic contacts in the cleft near SUb1, we substituted anthracene or phenanthrene to the naphthalene position. Results for both substitutions show more favorable docking scores than for 3k, with anthracene showing slightly better performance than phenanthrene (Figure 9). The favorable contacts arise from interaction with Asp166, a residue that rotates freely in the CoV2-3k simulations, indicating that it may be available to form a stable interaction with ligands capable of reaching it. Additionally, both the anthracene and phenanthrene-substituted ligands maintained all the other essential interactions we identified for 3k. In an attempt to increase polar interactions, we added a hydroxyl to the naphthalene moiety to form a hydrogen bond with Gly266 or other hydrogen bond acceptors in the area. We found that the hydrogen bond with Gly266 does indeed form as expected, with a distance of just 1.74 Å. However, in this conformation, the important hydrogen bonding group on the ligand that usually interacts with Asp164 is slightly out of position (Figure 9). Despite this, the binding is still more favorable than that of 3k. Also, notably, our MD simulations of CoV2-3k that started without the 3k-Asp164 hydrogen bond quickly formed that hydrogen bond after the simulation began, providing evidence that the same will likely happen in the case of the new ligand. Taken altogether, the MD and docking results show that the 3k scaffold should be capable of exploiting bulkier hydrophobic groups or polar groups at the naphthalene end to establish both favorable hydrophobic and polar contacts while maintaining the essential residue interactions that made 3k successful in CoV1. These are but a few of many possible enhancements to the naphthalene-based scaffold, and they require further validation through MD simulation or experimental assays. However, this serves as a strong proof-of-concept for future CoV2 PLpro design directions.

Pair-wise force distribution analysis (Supplementary Figure S11) and interaction energies (Supplementary Table S1) indicate that the binding mode of 3k in CoV2 is dependent on both strong vdW interactions and hydrogen bonds (Supplementary Figure S13), highly similar to that of 3k in CoV1 (Table 2). The interaction with Tyr268 is a dominant one in all ligand-bound simulations. The residue engages in a hydrogen bond donated by the amide nitrogen of 3k and a T-shaped pi-stacking interaction with the naphthalene moiety (Figure 8), which is seen with all naphthalene-based inhibitors (Baez-Santos et al., 2015). The hydrogen bond between 3k and Asp164 (Figure 1C) is another strong protein-ligand interaction shared in CoV1 and CoV2. All major ligand interactions with binding site residues are shown in Supplementary Figure S11 and listed in Supplementary Table S1.

In addition to 3k, 6-mercaptopurine (6MP) from the thiopurine class of inhibitors has been reported to reversibly inhibit CoV1 (Chou et al., 2008) and MERS-CoV (Cheng et al., 2015) PLpro activity. To assess its potential for inhibiting CoV2 PLpro, we docked 6MP in the active site of the enzyme and the putative ligand binding site (Figure 1A) based on the proposed binding poses from existing studies. We ran three independent simulations of the two complexes for 200 ns each (MD7a and MD7b). The compound dissociated from the putative ligand binding site within 80 ns or less and no stable intermolecular interactions were established. The compound stayed within the active site during two of the three MD7b simulations, however, it remained highly mobile and unstable in the pocket (Supplementary Figure S14). Because the ligand was unstable in both binding sites, we did not compute interaction energies between 6MP and Cov2 PLpro. Our analysis suggests that ligand 6MP is a weak binder and may be a poor inhibitor of Cov2 PLpro.

## Conclusion

By analysing the dynamics of ligand-free and ligand-bound CoV2 PLpro, we have gained insight to the important dynamics and intramolecular interactions relevant to its function and the development of small molecule inhibitory drugs. The BL2 loop, zinc binding region, and UBL domain are the most mobile protein regions, and CoV2 PLpro overall dynamics are extremely similar to those of CoV1 PLpro. SUb1 contains hydrophobic residues that contact the ligand in the 3k binding site, while SUb2 is adjacent to the highly mobile UBL domain and is affected by contacts to its Ub-interacting residues brought about by UBL domain rotation.

We docked two ligands, 3k and 6MP, known to inhibit CoV1 and MERS-CoV PLpro, respectively, into CoV2 PLpro to assess their ability as CoV2 inhibitors and identify opportunities for further optimization of the ligand scaffolds. We found that not only can 3k bind strongly to CoV2 PLpro, but that there is room for further optimization of binding affinity by exploitation of space in the small hydrophobic cleft near Pro247, Pro248, and Tyr264, or by making additional residue contacts in the open pocket region at the opposite end of the binding site. By docking newly designed ligands based on 3k, we validated our optimization suggestions, which showed better docking scores than 3k and exhibited binding modes in agreement with our proposed concepts that still maintained the intermolecular interactions that characterize successful naphthalene-based inhibitors. 6MP was unable to bind stably in the 3k site and dissociated quickly in all three simulations. It associated for longer to the active site, however, even when it remained bound, the compound was unstable.

Our results show that naphthalene-based inhibitors or similar compounds should have an inhibitory effect on CoV2 PLpro, and we have provided detailed suggestions for how this ligand scaffold can be furthered improved by engaging residues in underutilized space of the binding site. This study also generates an ensemble of CoV2 PLpro conformations that illustrate potential inhibitor-protein interactions for structure-based inhibitor design and elucidates protein dynamics relevant to Ub or Ub-like protein binding.

## Materials and Methods

### MD Simulation Protocol

MD simulations were prepared and run using the *Amber18* molecular dynamics package with GPU acceleration (Götz et al., 2012; Case et al., 2018). Force fields ff14SB (Maier et al., 2015) and the general Amber force field (GAFF2) (Wang et al., 2004) were used on proteins and ligands, respectively. Ligands 3k are 6MP were parameterized using Amber’s *antechamber* program with the AM1-BCC charge assignment method (Jakalian et al., 2002). All systems were solvated with a rectangular box of explicit TIP3P water extending 12 Å beyond the solute edges, and each contained no more than 1–3 Na^+^ or Cl^–^ counterions, which were added only to neutralize overall system charge. Systems were minimized in four steps. First, using Generalized Born implicit solvent (Chen et al., 2008), we minimized the hydrogen atoms, then protein sidechains, and finally the entire protein for 500, 1000, and 5000 steps, respectively. Next, the entire solvated structure was minimized for 5000 steps. Solvated systems were equilibrated in the isothermic-isobaric (NPT) ensemble from 50 to 275 K in 25 K increments for 100 ps each, and finally at 298 K for 500 ps. Production simulations were performed in the NPT ensemble at 298K using the Langevin thermostat with a 2 fs timestep. A 12 Å cutoff distance was used for direct non-bonded energy calculations and long-range electrostatics were calculated by the particle mesh Ewald method (Sagui et al., 2003). The SHAKE algorithm (Ryckaert et al., 1977) was employed to constrain all bonds involving hydrogen. Raw trajectories were saved every 2 ps and then processed using Amber’s *cpptraj* (Roe and Cheatham, 2013) for analysis.

### Selection of Initial Structures for MD Simulation

Initial coordinates for CoV2 PLpro simulations were obtained from two crystal structures of the ligand-free protein, PDB IDs 6W9C (Osipiuk et al., 2020b) and 6WRH (Osipiuk et al., 2020a). The ligand-bound complexes for CoV2 were obtained by docking ligands into a protein conformation selected from MD1; details are provided in the following subsection. CoV1 PLpro simulations began from a crystal structure of a 3k-bound complex, PDB 4OW0 (Baez-Santos et al., 2014). Ligand 3k was manually removed from the binding site for our ligand-free CoV1 PLpro simulation. MERS-CoV PLpro was simulated only in the ligand-free state, starting from crystal structure 4RNA (Lee et al., 2015). For simplicity, we have indexed these simulations as shown in Table 1.

### Ligand Docking to CoV2 PLpro

Force distribution analysis tool (FDA) (Stacklies et al., 2011) was used to identify the residues interacting with 3k in CoV1 PLpro (Supplementary Figure S15), and since these residues are identical in CoV2 PLpro, we used them as a ligand docking site. To choose a CoV2 PLpro conformation that was highly similar to the minimized CoV1 3k-bound crystal structure, we found the CoV2 frame from MD1-1 with minimum RMSD between key binding site residues to use for docking (Supplementary Figure S15). The ligands were docked to this single PLpro conformation using Molecular Operating Environment (MOE) (2018). Four poses (Figure 7) of 3k in this site were selected for MD simulations. To obtain poses A and D, we used the induced fit docking option with constrained/tethered side chain rotations allowed; poses B and C were obtained using the same induced fit option with free sidechain rotation allowed. Poses A and B closely resemble the Cov1 PLpro–3k crystal structure, PDB 4OW0. Pose C is a rotamer of A with a 180° rotation of the naphthalene moiety, while in pose D the piperidine moiety is rotated 180° with respect to A. In addition to the 3k binding site, 6MP was also docked to the active site following the same method. The designed ligands reflecting our suggested modifications to the 3k scaffold were docked in the 3k site by the same protocol as above to the same CoV2 PLpro conformation as 3k.

### Simulation Analysis

#### Trajectory Visualization and Dihedral Analysis

The simulations were visualized using Visual Molecular Dynamics (VMD) (Humphrey et al., 1996) and *MOE*. Dihedral angle populations and entropy were calculated using *T-Analyst* (Ai et al., 2010). MD simulations trajectories have been made available on our group website: http://chemcha-gpu0.ucr.edu/software/ and are deposited at the COVID-19 Molecular Structure and Therapeutics Hub: https://covid.molssi.org/. The trajectories there have been stripped of water and counterions and were saved every 10 ps. Trajectories with water are available upon request.

#### Cartesian Principal Component Analysis

To observe major protein motions, we performed principal component analysis (Hotelling, 1992, 1933) of α-carbon atoms in the 1 μs trajectory of ligand-free CoV2 PLpro. PCA reduces the high-dimensional data set of all α-carbon motions throughout the MD trajectory to its principal components (PCs), the directions which contain the largest motions. We used the average α-carbon positions as references. The first and second largest PCs were analyzed to reveal the dominant motions.

#### MM/PBSA

We used the MM/PBSA method (Wang et al., 2018) to evaluate the intermolecular interactions between ligands and PLpro. From a total of 20,000 MD frames making up the 200 ns ligand-bound trajectories, system conformations were analyzed every 2 ns. This method computes the energy (E) of a system from the protein, ligand, and protein-ligand complex, and computes the interaction energy as Δ < E > = < E_*complex*_ > - < E_*protein*_ > - < E_*ligand*_ >. < E > denotes the computed average energy from a given MD trajectory. The default values of a solute dielectric of 1.0 and solvent dielectric of 80.0 were used. The total binding energy term was computed as E_*MM/PBSA*_ = E_*MM*_ + G_*PB*_ + G_*np*_, where E_*MM*_ includes standard molecular mechanics force field terms, G_*PB*_ is the solvation energy computed by solving the Poisson Boltzmann (PB) equation, and G_*np*_ is the non-polar energy estimated from the solvent accessible surface area (A) as γA + b + G_*disp*_. Here γ is the surface tension, b is a correction term, and G_*disp*_ is the free energy of forming attractive solute-solvent van der Waals interactions (Tan et al., 2007). In this work, γ = 0.03780 kcal mol^–1^ Å^–2^ and b = -0.5692 kcal mol^–1^.

### Experimental Assay

CoV2 PLpro gene was synthesized at GenScript (Piscataway, NJ), expressed in *Escherichia coli* and purified by hexahistidine tag followed by size exclusion chromatography. Compound 3k was synthesized and dissolved by 100% DMSO at 10 mM, then diluted to phosphate saline buffer to desired concentration (final DMSO is 1%). 1D NMR WaterLOGSY spectra of compound 3k at free and PLpro-mixed conditions were collected on a Bruker AVANCE III HD 600 MHz spectrometer equipped with a BBFO probe. Each 1D WaterLOGSY spectrum was acquired with a mixing time of 2 s and a relaxation delay of 2 s, and was referenced to 2,2-dimethyl-2-silapentane-5-sulfonate (DSS) at 0 ppm. The experiments were acquired at 298 K with concentrations of 3k and CoV2 PLpro at 100 and 5 μM, respectively.

## Data Availability Statement

All datasets generated for this study are included in the article/Supplementary Material.

## Author Contributions

YB ran and analyzed the simulations and produced the figures. TC ran and analyzed the simulations and wrote the manuscript. Y-CL collected and analyzed the NMR data. K-PW conceived the research, prepared the reagents, and analyzed the NMR data. All authors contributed to the article and approved the submitted version.

## Conflict of Interest

The authors declare that the research was conducted in the absence of any commercial or financial relationships that could be construed as a potential conflict of interest.
