# Supplementary material for: Insights Into Dynamics of Inhibitor and Ubiquitin-Like Protein Binding in SARS-CoV-2 Papain-Like Protease
Source: Front Mol Biosci. 2020 Aug 4;7:174. doi: 10.3389/fmolb.2020.00174 (PMC7417481; doi:10.3389/fmolb.2020.00174)
Supplement: Supplementary file 1 [file Table_1.docx]

***Supplementary Material***

***Supplementary Figures***

***
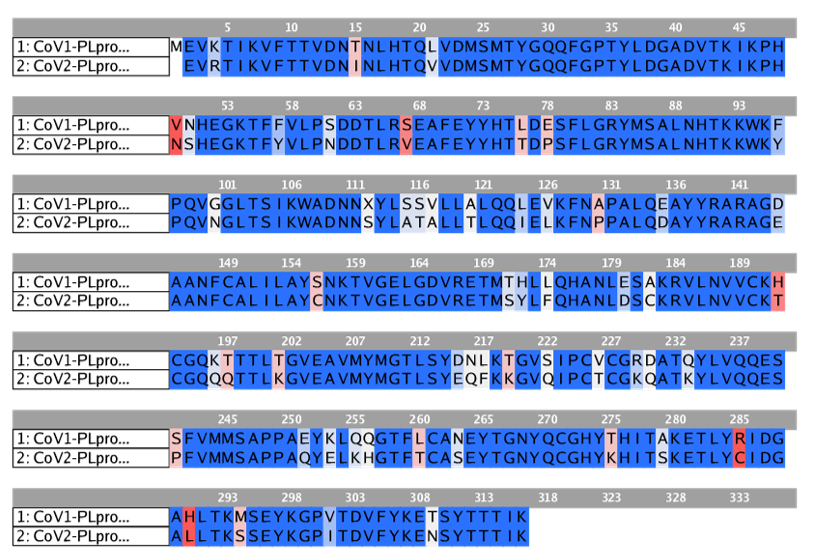
***

**Supplementary Figure 1.** Amino acid sequences of CoV1 and CoV2 PLpro.


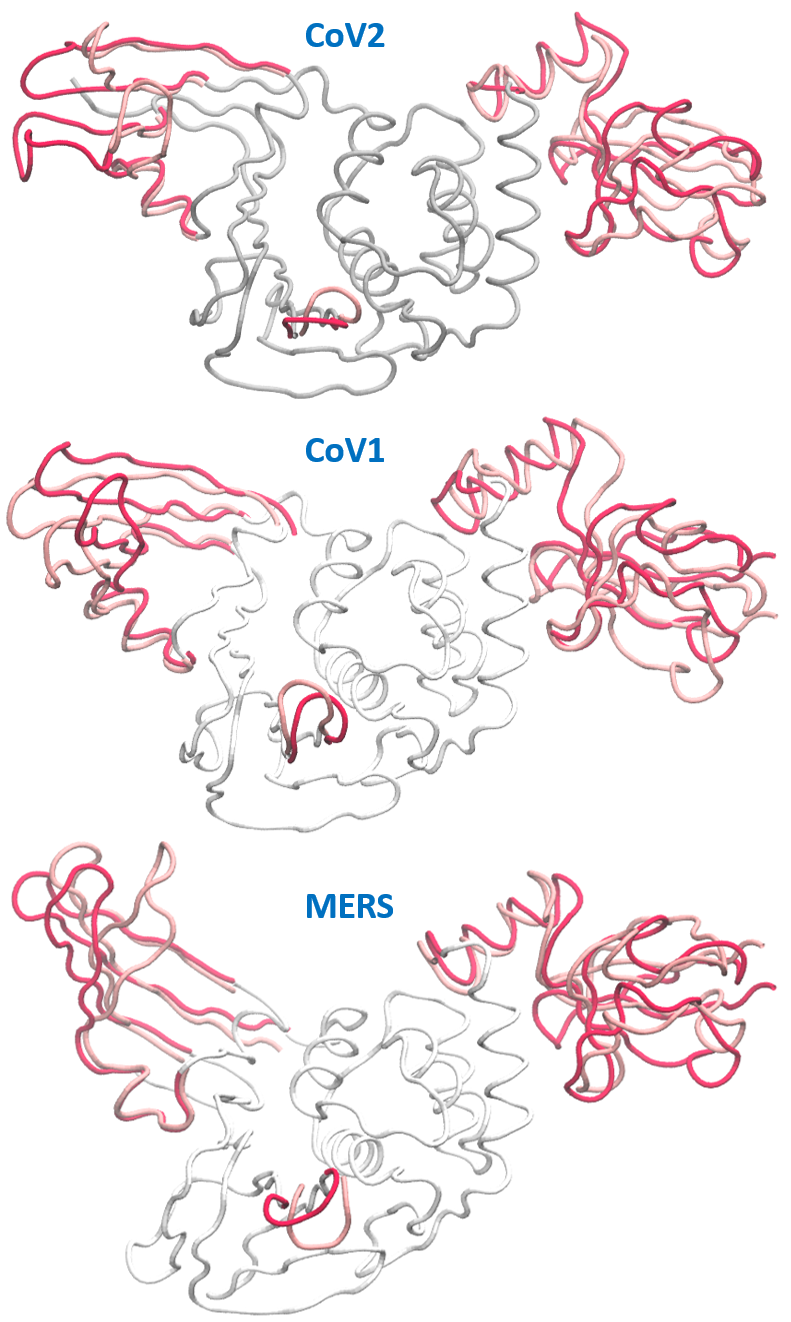


**Supplementary Figure 2.** PCA shows the most flexible regions of CoV2, Cov1 and MERS PLpro highlighted in red and pink to show the two extremes of a region’s range of motion (motion exaggerated 3-fold for clarity). From left to right, red/pink protein indicates the fingers domain, BL2 loop, and UBL domain.


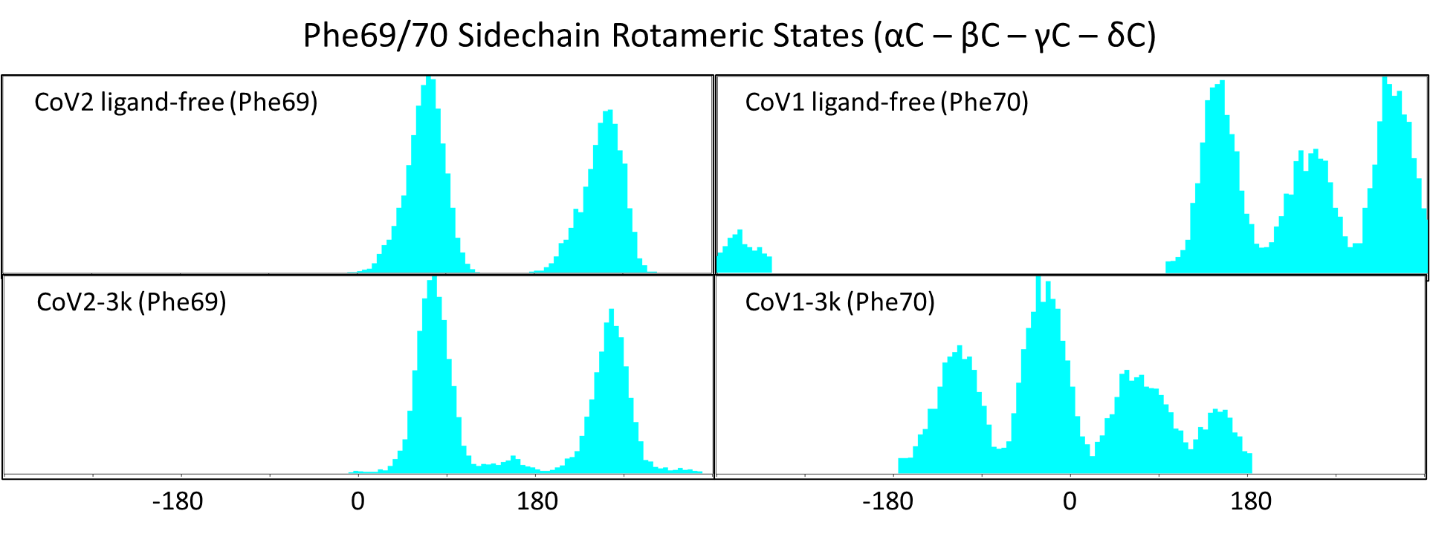


**Supplementary Figure 3.** Conformation populations of the Ub-interacting residue Phe69/70 sidechain in ligand-free and 3k-bound CoV2 and CoV1 PLpro. CoV2 shows only two conformations, whereas CoV1 shows four, which may cause unique interactions between CoV2 PLpro and Ub or Ub-like proteins at SUb2. This difference is seen in both ligand-free and -bound systems.


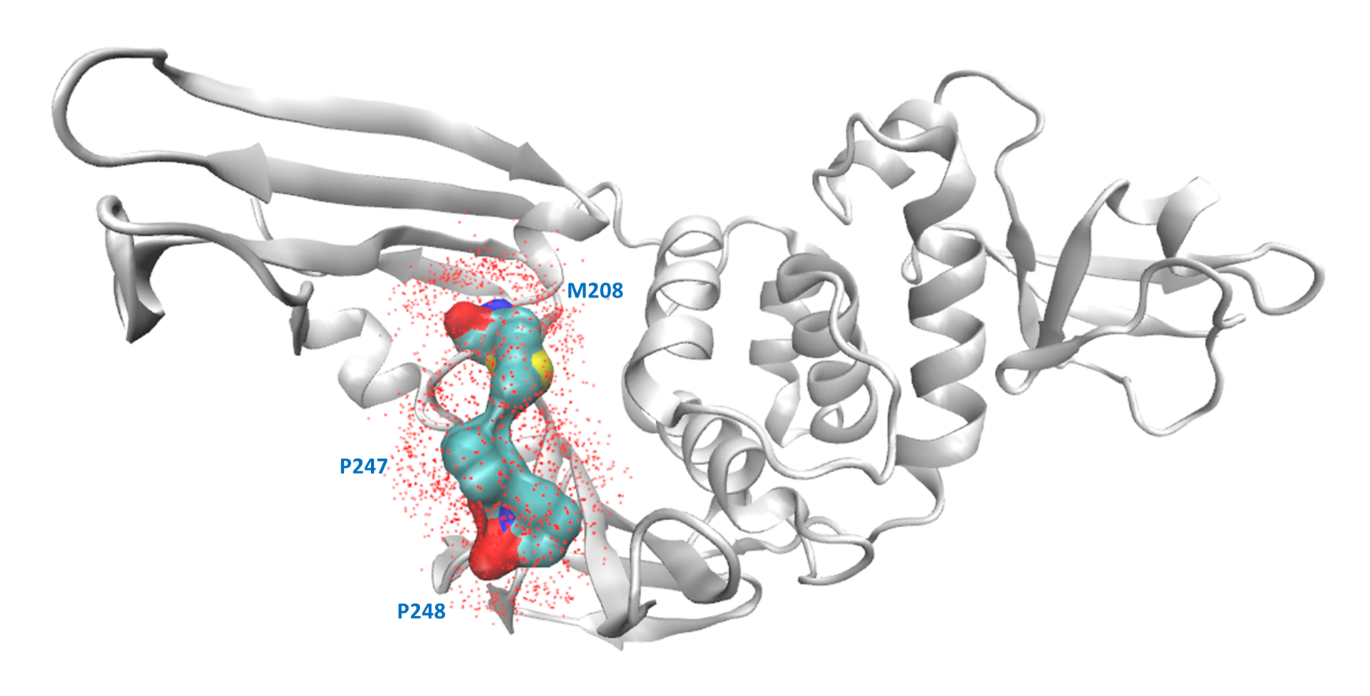


**Supplementary Figure 4.** The hydrophobic region of SUb1 (multi-color surface), which contains ligand-interacting residues M208, P247 and P248 in the putative bind site, is exposed to solvent in ligand-free systems. The residues here are available to establish new hydrophobic ligand interactions, which may be able to disrupt Ub or Ub-like protein binding at this site.


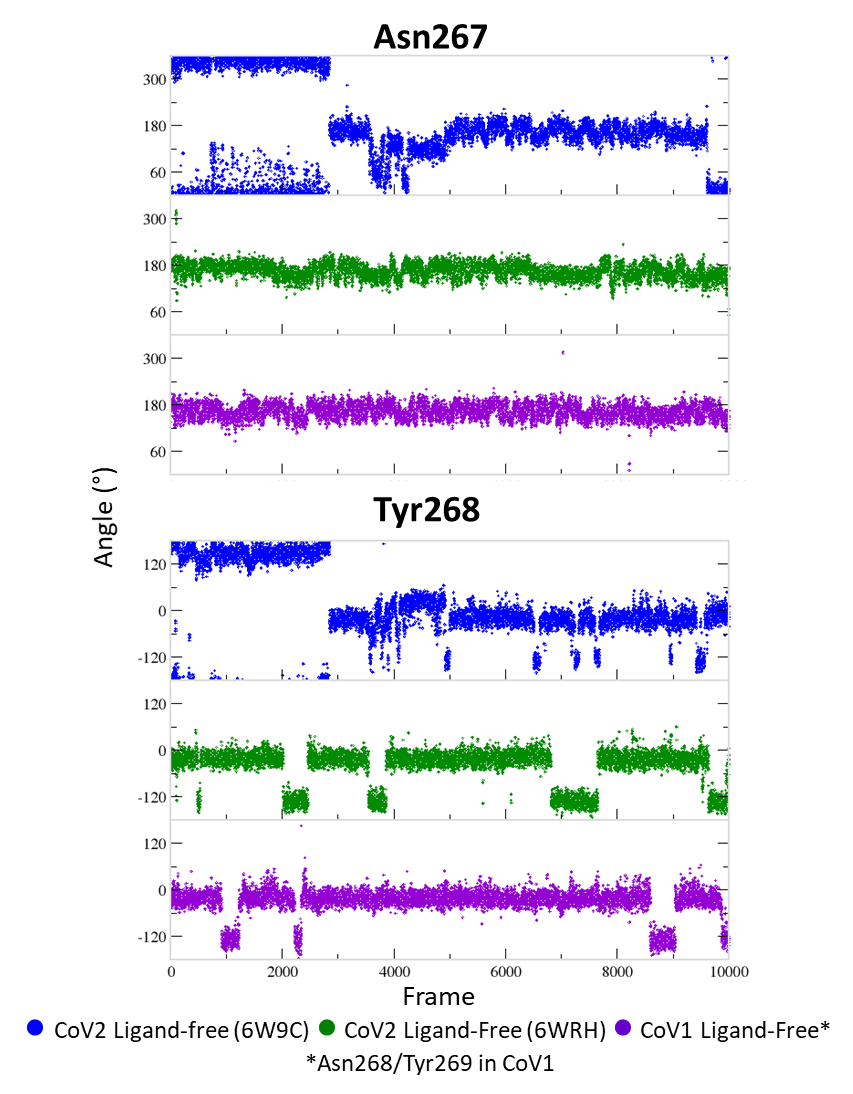


**Supplementary Figure 5.** Psi angle in BL2 loop residues Tyr268 and Asn267 over simulation time for ligand-free CoV2 PLpro starting from the two different CoV2 crystal structures, 6W9C and 6WRH, and in CoV1 PLpro. The unique outward conformation of these residue in 6W9C results in rotameric states of key ligand-interacting residue Tyr268 that may have implications on ligand binding in the putative site.


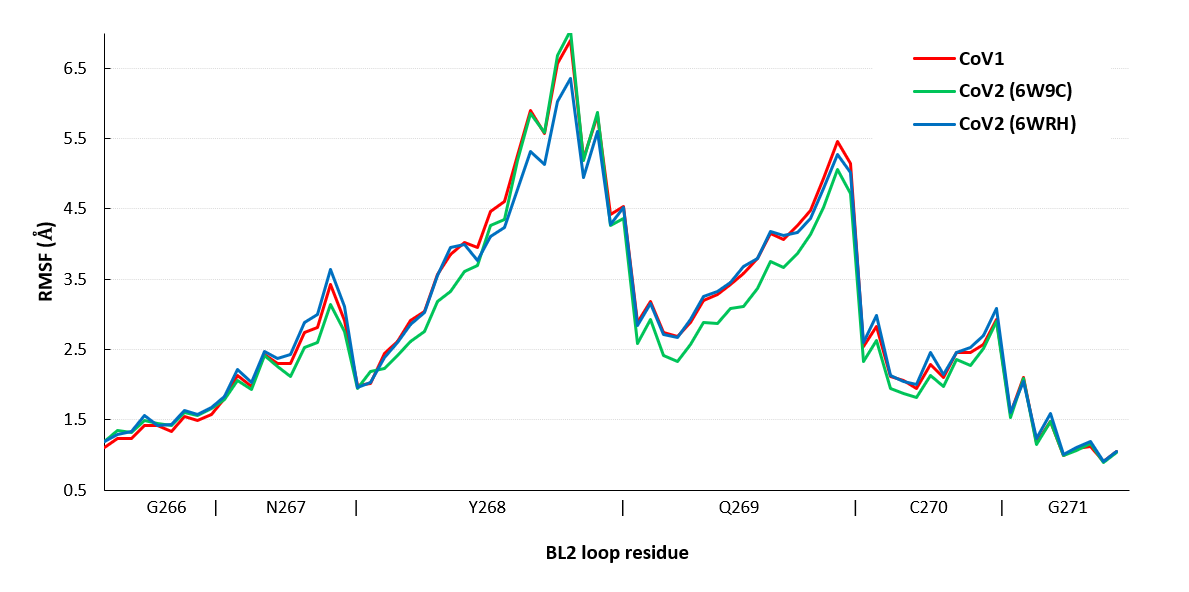


**Supplementary Figure 6.** Root-mean-square fluctuation (RMSF) for all atoms in the BL2 loop during only the last 500 ns of simulation of ligand-free CoV1 PLpro and the two CoV2 PLpro simulations beginning from different crystal structures (6W9C and 6WRH). In the last 500 ns of simulation, after the unique starting conformations of Asn267 and Tyr268 in crystal structure 6W9C go away, the dynamics of the loops in all three systems become highly similar.


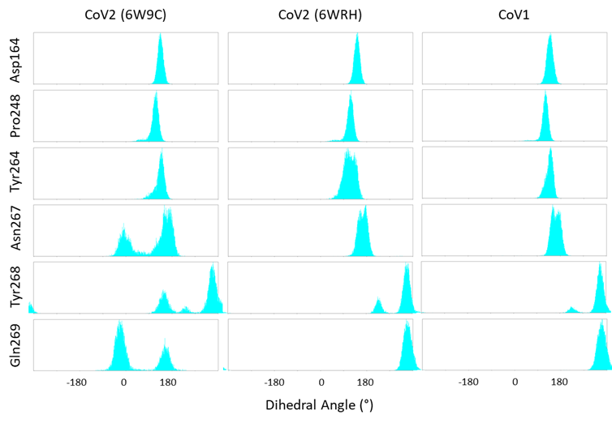


**Supplementary Figure 7.** Backbone dihedral angle populations of important binding pocket residues in ligand-free CoV1 and CoV2 PLpro.


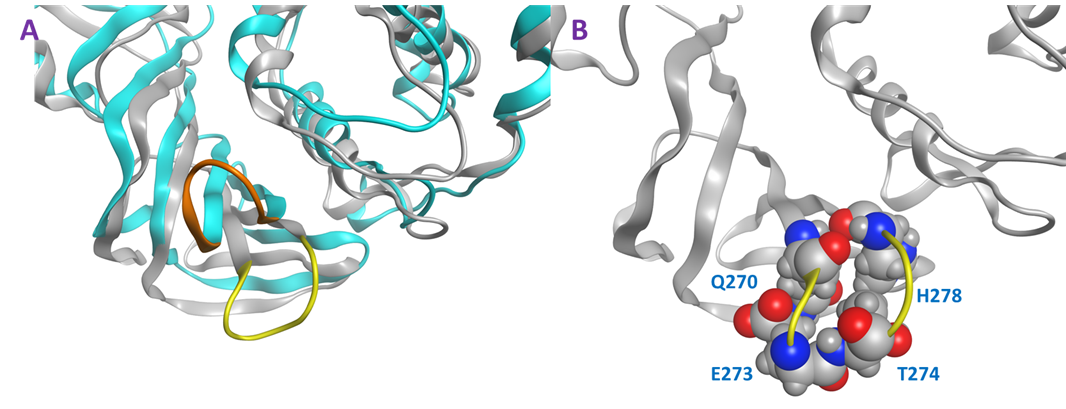


**Supplementary Figure 8.** Depiction of the open conformation of the MERS-CoV BL2 loop a) BL2 loop of MERS-CoV PLpro (yellow) compared to CoV2 PLpro (orange) showing the more open conformation in MERS-CoV. b) Hydrophobic interactions between the labeled residues (shown as van der Waals spheres) hold the MERS-CoV BL2 loop in the open conformation.


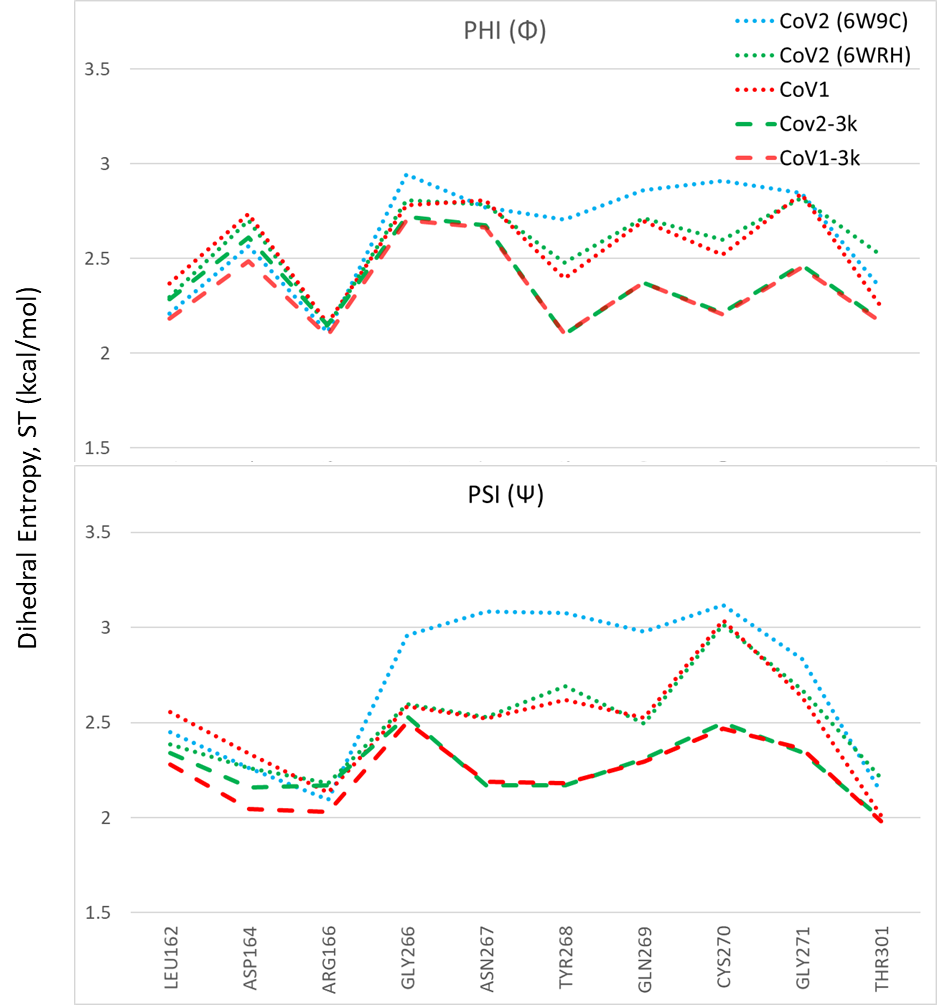


**Supplementary Figure 9.** Dihedral entropy calculated at 298 K for phi and psi torsions of ligand-interacting residues in ligand-free and 3k-bound CoV1 and CoV2 PLpro.


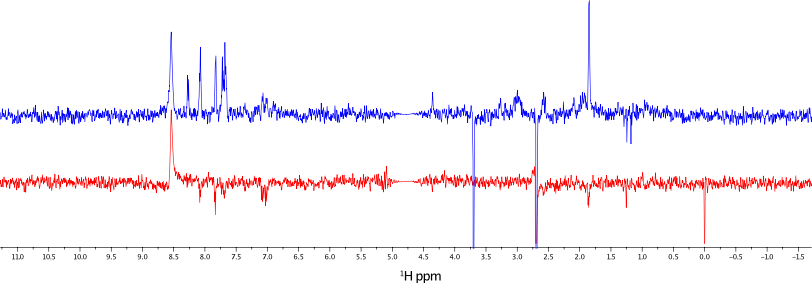


**Supplementary Figure 10.** NMR WaterLOGSY spectra of 3k alone (red) and mixed with 5 µM CoV-2 PLpro (blue). The positive peaks in the mixed sample suggest aromatic protons of 3k interact with PLpro. 0.0 ppm in the 3k-alone spectrum indicates internal reference (DSS). Peaks at 2.7 and 3.7 ppm are signals of 1% DMSO and 0.2 mM Tris, respectively.


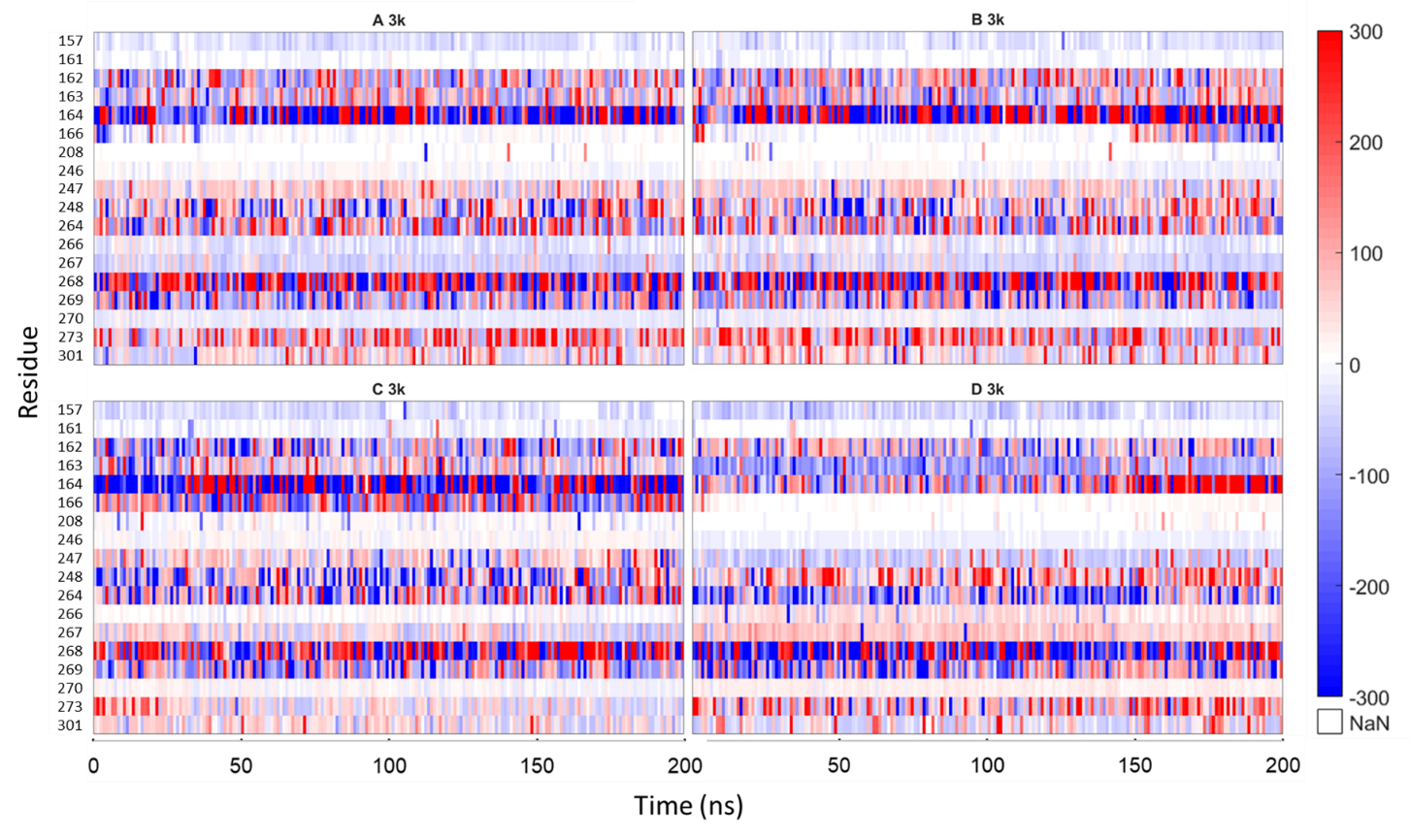


**Supplementary Figure 11.** Residue-wise protein-ligand interaction forces, where blue indicates attractive forces and red indicates repulsion, from one 200 ns CoV2 PLpro-3k complex simulation beginning from each of the four binding poses, A-D. The interactions with residues Tyr268 and Asp164 are dominant for all four. Pose D, despite starting with its hydrogen bond donor pointed away from Asp164, establishes this important interaction in the latter half of the simulation. Force values are in piconewtons.


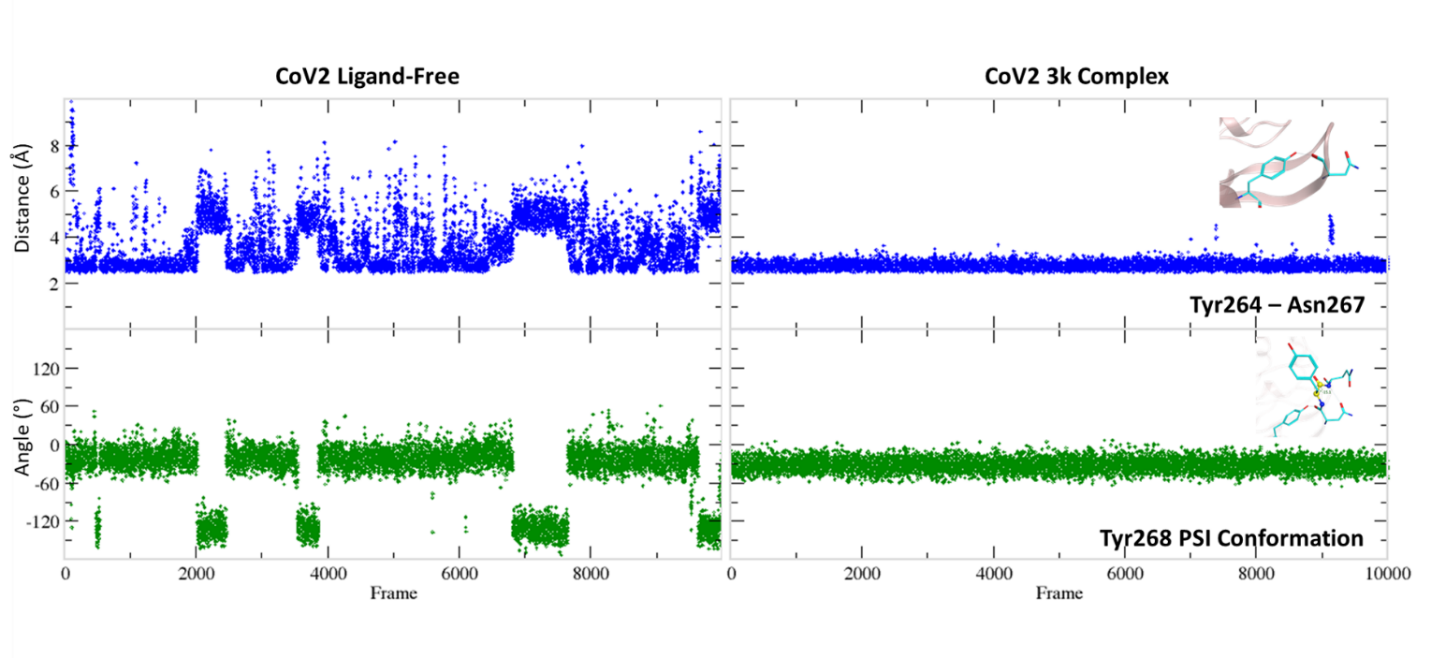


**Supplementary Figure 12.** Tyr264-Asn267 hydrogen bond length and Tyr268 psi angle over time. In ligand-free CoV2 PLpro (left), a short hydrogen bond length between Tyr264 and Asn267 (top, blue) has a strong correlation with a closed BL2 loop conformation (bottom, green). After ligand binding (right), this distance is consistently short and the loop conformation remains closed.


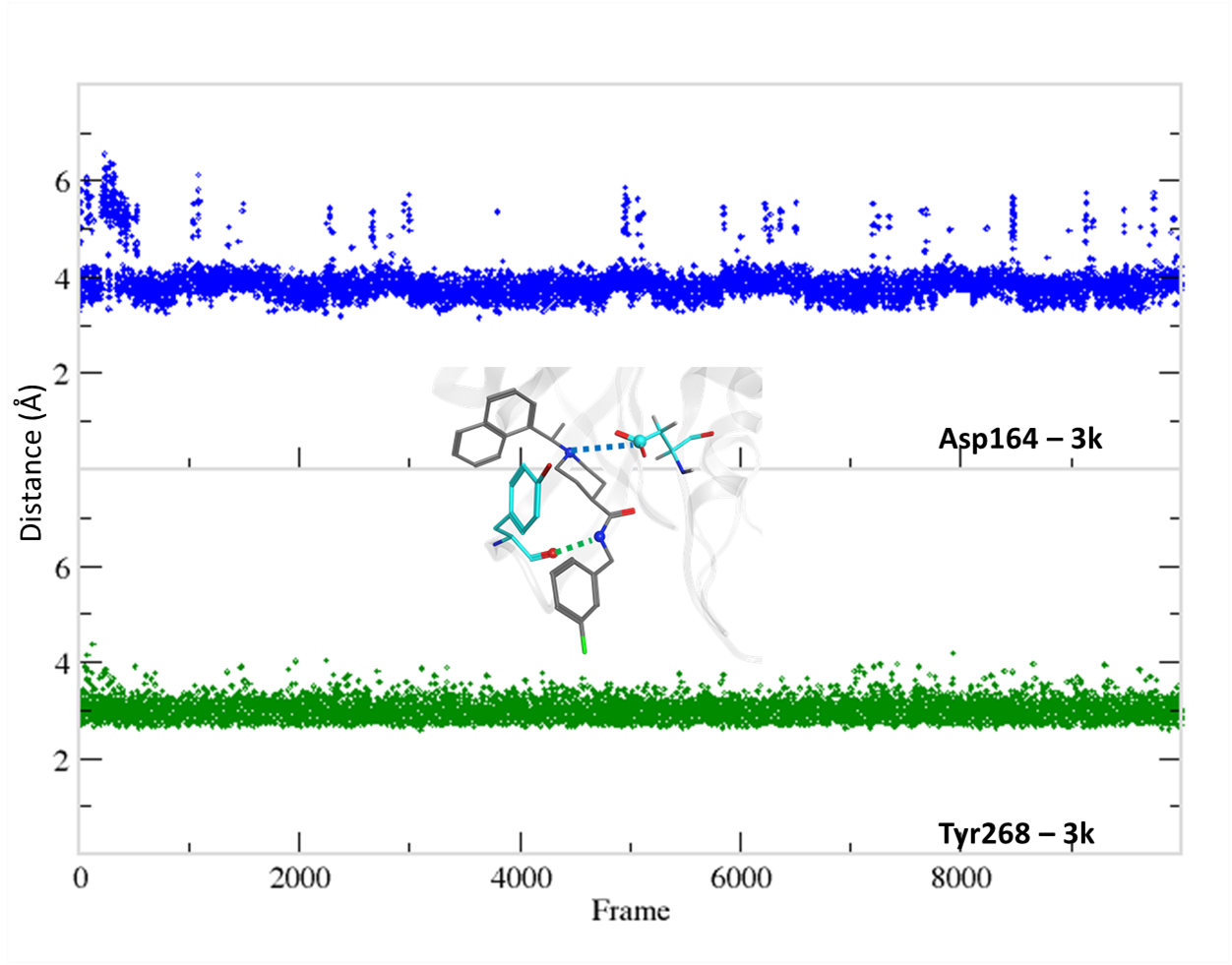


**Supplementary Figure 13.** Distance of the two key ligand-protein hydrogen bonds between 3k and Asp164 or Tyr268 over 200 ns of simulation. The minimal fluctuation in distance indicates the stability of the bonds and the very limited space in this part of the binding pocket.


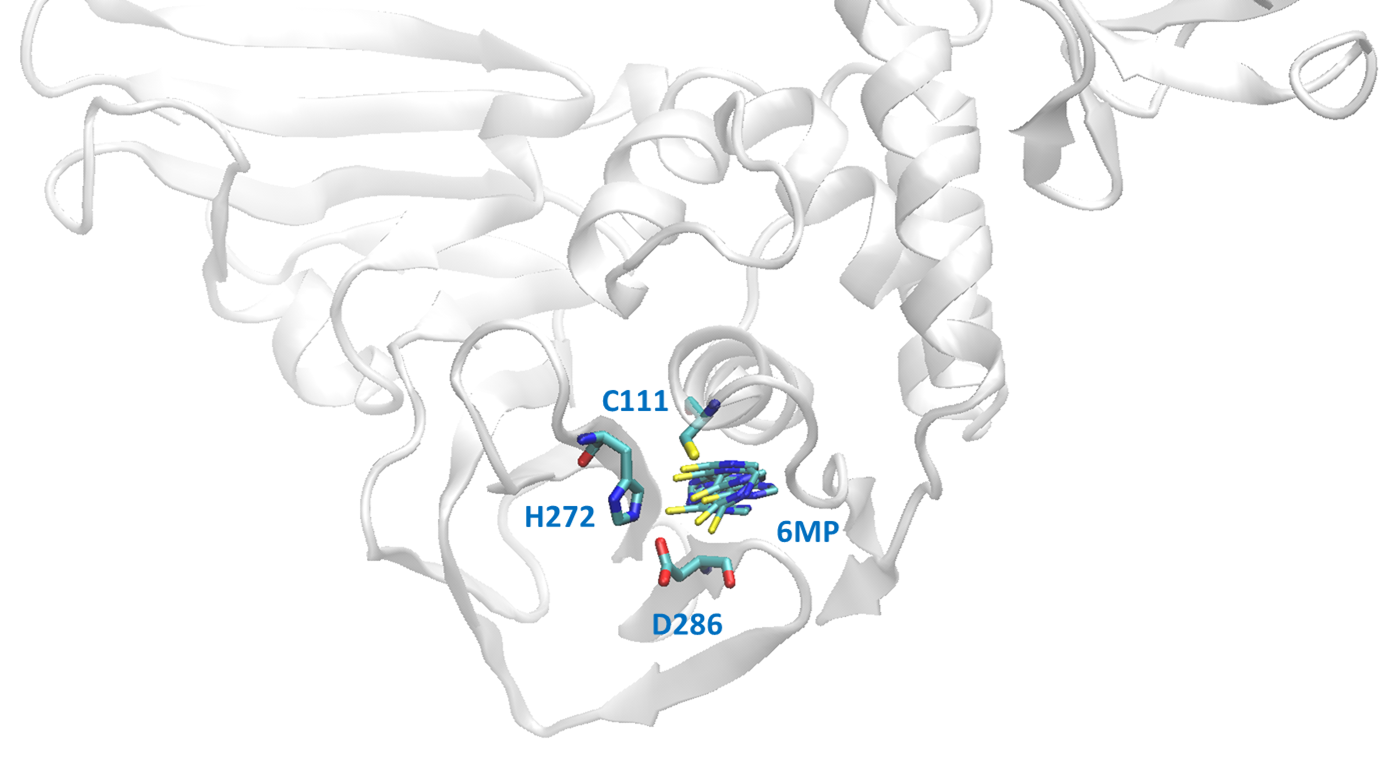


**Supplementary Figure 14.** Several conformations, shown as superimposed MD simulation frames, were observed for ligand 6MP in the active site of CoV2 PLpro, indicating it is a weak binder and does not appear to be a promising inhibitor.


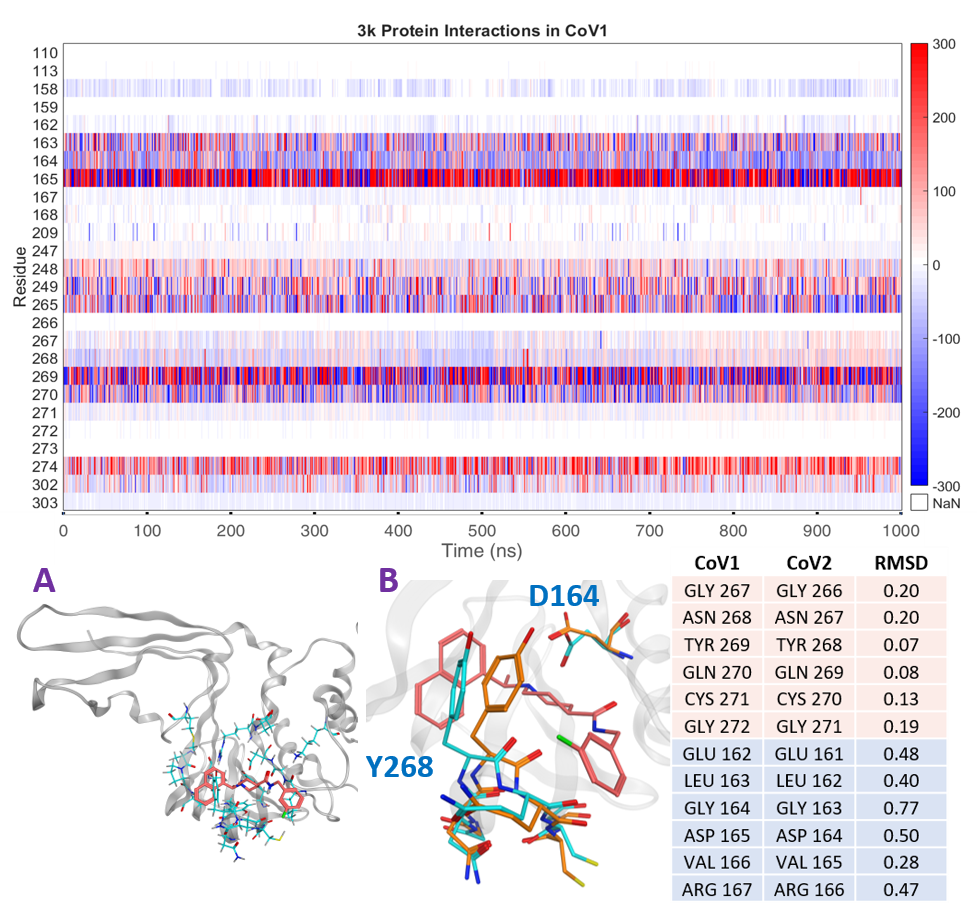


**Supplementary Figure 15.** Residues selected as the docking site. Top) Residue-wise force distribution heatmap indicates protein residues interacting with ligand 3k in CoV1 PLpro. c) The docking site residues selected based on force analysis displayed in CoV2 PLpro. b) Conformation of key residues in CoV1 PLpro (orange) and the conformation of the same residues selected for docking in CoV2 PLpro (teal). Bottom right: table of RMSD values between binding site residues in the minimized CoV1 PLpro crystal structure and CoV2 PLpro conformation used for docking. Force values are in piconewtons.

|  | CoV2 - pose A | | | CoV2 - pose B | | | CoV2 - pose C | | |
| --- | --- | --- | --- | --- | --- | --- | --- | --- | --- |
| Res | **Total** | **vdW** | **elec** | **Total** | **vdW** | **elec** | **Total** | **vdW** | **elec** |
| 164 | -77.7 ± 16.1 | -2.4 | -75.4 | -74.6 ± 9.8 | -3.03 | -71.6 | -83.5 ± 5.8 | -2.5 | -81.0 |
| 268 | -21.5 ± 2.1 | -9.6 | -11.9 | -21.6 ± 2.0 | -9.5 | -12.1 | -21.1 ± 2.0 | -9.6 | -11.6 |
| 167 | -16.6 ± 12.5 | -0.2 | -16.4 | -18.3 ± 10.6 | -0.2 | -18.0 | -16.5 ± 12.1 | -0.2 | -16.3 |
| 267 | -7.4 ± 7.2 | -1.5 | -5.9 | -7.9 ± 8.1 | -1.3 | -6.5 | -5.8 ± 9.4 | -1.3 | -4.5 |
| 248 | -7.3 ± 3.2 | -3.8 | -3.5 | -7.5 ± 3.0 | -3.9 | -3.5 | -7.4 ± 3.0 | -3.4 | -3.9 |
| 163 | -5.5 ± 3.4 | -1.9 | -3.5 | -4.5 ± 3.1 | -1.8 | -2.7 | -3.2 ± 3.16 | -2.0 | -1.2 |
| 269 | -3.9 ± 10.7 | -4.4 | 0.6 | -2.7 ± 10.4 | -4.5 | 1.9 | -5.5 ± 11.2 | -4.2 | -1.2 |
| 249 | -3.0 ± 2.7 | -0.1 | -2.9 | -2.7 ± 3.2 | -0.1 | -2.6 | -3.3 ± 2.8 | -0.1 | -3.3 |
| 274 | 3.7 ± 8.5 | -0.0 | 3.8 | 7.5 ± 7.7 | -0.0 | 7.5 | 7.2 ± 7.1 | -0.0 | 7.2 |
| 165 | 5.3 ± 9.3 | -0.22 | 6.0 | 2.0 ± 8.8 | -0.3 | 2.4 | 3.14 ± 8.2 | -0.2 | 3.3 |
|  |  |  |  |  |  |  |  |  |  |
|  | **CoV2 - pose D** | | | **CoV1** | | |  | | |
| Res | **Total** | **vdW** | **elec** | **Total** | **vdW** | **elec** |  |  |  |
| 164 | -56.3 ± 14.7 | -3.3 | -53.0 | -74.3 6.8 | -3.2 | -71.1 |  |  |  |
| 268 | -23.7 ± 2.8 | -10.2 | -13.5 | -20.7 ±2.3 | -9.4 | -11.3 |  |  |  |
| 167 | -15.5 ± 12.2 | -0.2 | -15.2 | -19.1 ± 9.8 | -0.3 | -18.8 |  |  |  |
| 267 | -5.5 ± 8.0 | -1.6 | -3.9 | -7.8 ± 7.3 | -1.4 | -6.3 |  |  |  |
| 248 | -7.2 ± 2.6 | -4.0 | -3.2 | -7.4 ± 2.9 | -4.0 | -3.5 |  |  |  |
| 163 | -6.8 ± 4.0 | -2.0 | -4.9 | -6.2 ± 2.7 | -1.9 | -4.4 |  |  |  |
| 269 | -4.9 ± 7.5 | -4.6 | -0.3 | -3.1 ± 2.8 | -4.6 | 1.5 |  |  |  |
| 249 | -2.5 ± 2.9 | -0.1 | -2.4 | -3.1 ± 10.1 | -0.1 | -3.0 |  |  |  |
| 274 | 8.0 ± 9.2 | -0.0 | 8.0 | 4.2 ± 6.7 | -0.0 | 4.2 |  |  |  |
| 165 | 3.8 ± 7.5 | -0.3 | 4.2 | 8.3 ± 6.2 | -0.4 | 8.7 |  |  |  |

**Supplementary Table 1.** Total, electrostatic (elec), and van der Waals (vdW) residue-wise protein-ligand interaction energies from 3k-bound CoV2 and CoV1 PLpro simulations, in kcal/mol. Energies were calculated every 200 ps and then averaged over the 200 ns trajectory. Values are ± SD.
